# Associations of SAA1 gene polymorphism with Lipid lelvels and osteoporosis in Chinese women

**DOI:** 10.1186/1476-511X-12-39

**Published:** 2013-03-22

**Authors:** Zheng-Ping Feng, Xiao-Yu Li, Rong Jiang, Hua-Cong Deng, Mei Yang, Qin Zhou, Wen-Jun Que, Jia Du

**Affiliations:** 1 Department of Endocrinology, the First Affiliated Hospital of Chongqing Medical University, Chongqing 400016, China; 2 Stem Cell and Tissue Engineering Laboratory, Chongqing Medical University, Chongqing 400016, China

**Keywords:** SAA1, Genetic polymorphism, Osteoporosis

## Abstract

**Background:**

The development of osteoporosis is associated with several risk factors, such as genetic polymorphisms and enviromental factors. This study assessed the correlation between SAA1 gene rs12218 polymorphism and HDL-C lelvels and osteoporosis in a population of Chinese women.

**Methods:**

A total of 387 postmenopausal female patients who were diagnosed with osteoporosis (case group) based on bone mineral density measurements via dual-energy x-ray absorptiometry and 307 females with no osteoporosis (control group) were included in this study. Correlations between SAA1 gene rs12218 polymorphism and osteoporosis and HDL-C level were investigated through the identification of SAA1 gene rs12218 polymorphism genotypes using the polymerase chain reaction-restriction fragment length polymorphism (PCR-RFLP).

**Results:**

The TT genotype of rs12218 was more frequently in osteoporosis patients than in control subjects (P <0.001). And the rs12218 was found to be associated with plasma TG, HDL-C, LDL-C, and BMD levels in osteoporosis patients (P<0.05).

**Conclusions:**

The present results indicate that both osteoporosis and lipids levels are associated with the TT genotype of rs12218 in the human SAA1 gene.

## Background

Osteoporosis is a major public health problem with growing prevalence. Obesity and hyperlipidemia have been demonstrated to be closely related with osteoporosis [1-3]. And osteoporosis is especially prevalent in the elderly population, and it is a significant public health issue that reduces patient functioning and quality of life. Moreover, both osteoporosis and obesity have high genetic predisposition and the genetic correlation between them have been established across different ethnic groups [1,4]. Serum amyloid A (SAA) is a kind of apolipoprotein and is primarily synthesized in the liver by activated monocytes and macrophages [5]. As an apolipoprotein, SAA is associated with HDL-C and during inflammation can contribute up to 80% of its apoprotein composition [6]. Many studies have demonstrated that sustained high expression of SAA may contribute to atherogenesis [7,8], and that an elevated concentration of SAA is associated with an increased risk of CVD [9]. And serveral studies indicated rs12218 in the SAA1 gene was associted with carotid atherosclerosis [10] and peripheral arterial disease [11]. However, the relationships between SAA gene polymorphisms and osteoporosis remain unclear.

In the present study, we aim to study the relationship between SAA1 gene polymorphsim (rs12218) and HDL-C level and osteoporosis.

## Results and discussion

Table 1 shows the clinical characteristics of the study participants, the following values were significantly different between the 2 groups: systolic blood and age. There was no significant difference in the following variables between the 2 groups: DBP, body mass index (BMI), plasma concentration of total cholesterol (TC), plasma concentration of TG, HDL-C and LDL-C.

**Table 1:** Characteristics of these two groups

	**Control group**	**Osteoporosis group**	**P value**
Subjects (n)	387	307	
Age (years)	51.32±4.618	55.45±8.055	<0.001
BMI (kg/m2)	24.17±3.15	24.22±3.82	0.827
SBP (mmHg)	119.35±10.75	117.47±9.98	0.019
SBP (mmHg)	74.93±8.23	75.66±8.37	0.245
TG (mmol/L)	1.02±.44	1.05±.46	0.372
TC (mmol/L)	4.17±.95	4.16±.95	0.861
HDL-C (mmol/L)	1.28±.51	1.24±.40	0.365
LDL-C (mmol/L)	2.49±.73	2.54±.82	0.443

Table 2 shows the distribution of the genotypes and alleles of the rs12218. The genotype distribution of each rs12218 did not show significant difference from the Hardy-Weinberg equilibrium values (data not shown). For total participants, the genotype and the allele distribution of rs12218 differed significantly between the osteoporosis patients and the control participants (both P<0.001). The TT genotype and T allele were more common in the osteoporosis patients than in the control participants. Logistic regression was performed with and without lipid disorders and other confounders. The TT genotype of rs12218 still differed significantly between these two groups (P<0.001, OR=7.610, 95% CI: 3.484-16.620, Table 3).

**Table 2:** Distributoion of genotypes and allels

	**Genotypes**				**Allel**		
	**CC**	**CT**	**TT**	**P value**	**C**	**T**	**P value**
Osteoporosis group	46 (15.0)	79 (25.7)	182 (59.3)	<0.001	171	443	<0.001
Control group	9 (2.3)	128 (33.1)	250 (64.6)		146	628	

**Table 3:** Logistic regression analysis

	**B**	**S.E.**	**Wald**	**df**	**Sig.**	***OR *(95% CI)**
rs12218	2.029	0.399	25.927	1	<0.001	7.610(3.484-16.620)
age	0.134	0.017	60.331	1	<0.001	1.143(0.516-1.112)
BMI	0.045	0.026	2.991	1	0.084	1.047(1.105-1.183)
SBP	-0.049	0.010	25.591	1	<0.001	0.952(0.994-1.102)
DBP	0.053	0.012	20.468	1	<0.001	1.055(0.934-.970)
BUN	0.026	0.056	0.217	1	0.641	1.026(1.031-1.079)
GLU	0.044	0.189	0.055	1	0.815	1.045(0.920-1.145)
UA	0.001	0.001	1.033	1	0.309	1.001(0.721-1.515)
TG	0.078	0.207	0.143	1	0.706	1.081(0.999-1.004)
TC	-0.239	0.149	2.563	1	0.109	0.787(0.721-1.621)
LDL	0.191	0.181	1.110	1	0.292	1.210(0588–1.055)
Constant	-6.883	1.685	16.692	1	<0.001	0.001(.849-1.726)

**Figure 1:**
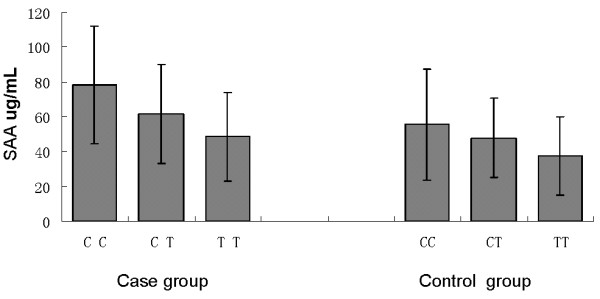
The SAA concentrations between genotypes of rs12218.

Table 4 shows the relationgship between rs12218 and TG, TC, HDL-C LDL-C, and BMD levels. In the osteoporosis group, we found that the rs12218 was not only significantly associated with plasma TC, HDL-C, and LDL-C levels (P=0.021, P=0.009, and P=0.009, respectively), but also associated with BMD (P<0.05). However, this association was not found in the control group. And, we did not find the TG level was significantly associated with rs12218 in these two groups. In addition, we also found the rs12218 was associated with the plasma SAA levels not only in the case group, but also in the control group (Figure 1).

**Table 4:** The relationship between genotypes and TG, TC, HDL, LDL and BMD

		**Osteoporosis group**			**Control group**		
	**Genotypes**	**N**	**(M±SD)**	**P value**	**N**	**(M±SD)**	**P value**
TG (mmol/L)	CC	9	0.77±.32	0.188	46	0.99±.46	0.603
	CT	128	1.01±.46		79	1.06±.44	
	TT	250	1.04±.44		182	1.07±.47	
TC(mmol/L)	CC	9	3.46±1.28	0.021	46	4.17±.94	0.933
	CT	128	4.07±1.03		79	4.12±.93	
	TT	250	4.24±.85		182	4.17±.96	
HDL(mmol/L)	CC	9	1.77±1.89	0.009	46	1.31±.45	0.325
	CT	128	1.23±.44		79	1.26±.36	
	TT	250	1.28±.42		182	1.22±.41	
LDL(mmol/L)	CC	9	1.94±.91	0.009	46	2.58±.72	0.524
	CT	128	2.40±.75		79	2.45±.76	
	TT	250	2.56±.71		182	2.57±.86	
BMD (L1-L4) (g/cm^2^)	CC	9	0.871±0.12	0.032	46	1.021±0.15	0.143
	CT	128	0.856±0.15		79	1.142±0.14	
	TT	250	0.814±0.14		182	1.014±0.15	
Hip BMD (g/cm^2^)	CC	9	0.679±0.09	0.047	46	0.875±0.11	0.092
	CT	128	0.653±0.11		79	0.851±0.13	
	TT	250	0.621±0.08		182	0.844±0.10	

In the present study**,** we found that variation in the SAA1 genes is associated with both osteoporosis and TC, HDL, and LDL levels in osteoporosis in Chinese population. This is the first study to observe the relationship btween SAA1 gene and osteoporosis. Osteoporosis is characterized by low bone mass, an increase in bone fragility, deterioration in bone microarchitecture, and an increase in the risk of fracture [12]. Some metabolic changes, such as those that occur due to a lack of estrogen, immobilization, metabolic acidosis, hyperparathyroidism, and systemic and local inflammatory diseases, affect the osteoclast count and activity associated with bone turnover [13]. Prostaglandins, insulin-like growth factors (IGFs), interleukins (IL-1, IL-6, and IL-11), tumor necrosis factor (TNF), and several local factors in bone, such as transforming growth factor (TGF), also contribute to the regulation of bone formation and resorption [13,14].

The gene for SAA1 was considered as a candidate for osteoporosis because it is the gene encoding one important inflammation factor, SAA, which is synthesized by the liver. A relationship between the SAA1 gene polymorphism and cardiovascular diseases has been reported previously [11,12,15]. Previous studies have investigated the SAA1 rs12218 polymorphism in the Chinese population, but its relationship with osteoporosis has not been investigated; this relationship was examined in our study for the first time. In the present study, the TT genotype of rs12218 significantly differed between osteoporosis patients and control participants, indicating that the risk of osteoporosis is increased in participants with the T allele of rs12218. Logistic regression analysis adjusted for some confounders showed that TT genotype distribution of rs12218 significantly differed between the osteoporosis patients and the control participants.

In additon, we also found the rs12218 significantly associated with the plasma TC levels in the osteoporosis patients, our finding was in line with Xie et al. reports. In the early 1970s, SAA was identified as the plasma protein responsible for forming tissue deposits called “amyloid (AA-type)” seen in diseases with underlying persistent acute inflammation [16,17]. Soon after its discovery, SAA was shown to be an acutephase protein produced by the liver within hours of tissue injury regardless of cause. Its plasma concentration can increase a 1000-fold within 24 h [18,19]. In plasma, SAA is associated with HDL [20,21] and, during severe inflammation, can contribute 80% of its apo-protein composition [22]. The displaced apoA-I is rapidly cleared by the liver and kidneys [23], together with a sharp decline in apoA-I gene expression during inflammation [24].

## Conclusions

In conclusion, the SAA1 gene polymorphism was associated with osteoporosis in Chinese population. And this association maybe related to the lipid disorder resulting from the SAA gene polymorphisms.

## Methods

### Subjects

Postmenopausal females who were admitted to the Department of Endocrinology, First Affiliated Hospital of Chongqing Medical University were informed of the study, and patients who opted for inclusion in the study were evaluated. Patients who were diagnosed with parathyroid, thyroid, liver, and rheumatological diseases that affect bone metabolism; patients with a history of malignancy or surgically induced menopause; and patients who used drugs affecting bone metabolism (e.g. corticosteroids, anticonvulsants, and heparin) during the clinical and laboratory assessments were excluded from the study. Erythrocyte sedimentation rate, complete blood count, serum alkaline phosphatase, calcium, phosphorous, serum glutamic oxaloacetic transaminase, serum glutamic pyruvic transaminase, gamma-glutamyl transpeptidase, blood urea nitrogen, creatinine, glucose, uric acid, albumin, total protein, urine calcium/creatinine, thyroid-stimulating hormone, parathyroid hormone, cortisol and vitamin D levels were measured prior to the study. A total of 694 patients satisfied the study criteria and were included in the study. The age, height, weight, and body mass index (BMI) of the participants were evaluated. All participants underwent dual-energy xray absorptiometry (DEXA) evaluations, and 387 postmenopausal females were diagnosed with osteoporosis based on this assessment (osteoporosis gorup). 307 patients without osteoporosis were included in the control group. All participants provided informed consent in compliance with the study protocol, which was approved by the Ethics Committee of the First Affiliated Hospital of Chongqing Medical University.

### Bone mineral density

The participants underwent DEXA scanning using a Hologic QDR 4500 W system (Hologic, Inc., Bedford, USA) to assess bone mineral density (BMD), and the lumbar spine (vertebrae L1-L4) and hip (femur neck) were evaluated. Patients with a mean bone density below 2.5 SD were diagnosed with osteoporosis, as recommended by the World Health Organization (WHO).

### Genotyping

Genomic DNA was extracted from peripheral blood leukocytes using a DNA extraction Kit (Beijing Bioteke Company Limited, Beijing, China). Genotyping was confirmed by polymerase chain reaction (PCR)-restriction fragment length polymorphism (RFLP) analysis. The primers of rs12218 were designed according to Xie’s protocol [10,11] as follows: Sense: 5^′^AACAGGGAGAATGGGAGGGTGGG3^′^; Antisense: 5^′^ GCAGGTCGGAAGTGATTGGGGTC3^′^. The PCR mixture was subjected to 35 cycles for 60 sec at 94°C, 30 sec at 54°C, and 40 sec at 72°C following the initial denaturation for 3 min at 94°C. These PCR products were digested by Bgl I restriction enzyme was according to manufacturer’s instructions. To ensure the results to be verified, we used sequenced genomic DNAs as positive controls in our assays.

### Statistical analysis

The data were evaluated using SPSS Version 17 software (IBM Corp. Armonk, New York, USA). The continuous variables were not normally distributed based on the Shapiro-Wilk test for normality. The Mann–Whitney U test was implemented for the comparison of the two groups. Medians (quartiles) are provided as descriptive statistics. The Pearson chi-square test was conducted for categorical variables. N and % values are provided. A p<0.05 was considered statistically significant.

## Abbreviations

SAA: Serum amyloid A; BMD: Bone mineral density; TG: Triglycerides; TC: Total cholesterol; HDL-C: High-density lipoprotein; LDL-C: Low-density lipoprotein.

## Competing interests

The authors declare that they have no competing interests.

## Authors’ contributions

ZPF carried out the molecular genetic studies and drafted the manuscript.XYL and RJ carried out the genotyping. ZPF and HCD participated in the design of the study and performed the statistical analysis. MY, QZ, JD and WJQ conceived of the study, and participated in its design and coordination and helped to draft the manuscript. All authors read and approved the final manuscript.
